# The Pathology of Daltonism

**Published:** 1882-01

**Authors:** 


### THE PATHOLOGY OF DALTONISM.

At the Society of Biology Dr. Galezowski read an interesting study upon this subject.

At a time when the examination of the question of vision in relation to color-blindness amongst railroad employees is the order of the day, it is well to know all the ocular alterations which can bring about this chromatic perturbation.

It is more than fifteen years since the author first drew attention to the traumatic troubles observed in different ocular and cerebral affections. To-day he brings new facts concerning the different modes of alteration of the color sense.

Syphilis, alcoholism, nicotinism, lead-poisoning, hysteria, locomotor ataxia, glycosuria, aphasia and hemiopia are so many pathological conditions which influence the sense of color.

Alcoholism is, as may be seen, one of the most frequent causes of the perturbation of sight. Alcoholic amblyopia exists without any lesion in the eye, but it may be accompanied by different chromatic troubles.

Of such amblyopes some cannot recognize color at all; some confound one color with another; others, finally, complain of successive contrasts of colors.

After having examined and fixed the color *red*, for example, and immediately after, *blue*, they do not see blue, but perhaps red, the first color which left an impression, or may be a mixture of red and blue, i. e., violet.

The abuse of tobacco also provokes very marked troubles of sight —amblyopia with central scotoma and chromatic perversions, similar in many respects to those of alcoholism. Nevertheless, M. Galezowski has never observed successive contrasts of colors.

Syphilis can reach all the membranes of the eye and induce visual troubles, of which some are due to central alterations with atrophy of the papilla and optical neuritis, and of which others are consequences of retinitis and choroiditis.

These affections injure, almost invariably, the sense of color, to a greater or less degree. In syphilitic retinitis there are chromatic perturbations relating to the tint or shade of colors. But in syphilitic choroiditis the perversion is almost constant, and often there is complete achromatopsia, or else the patient loses the perception of blue and yellow and recognizes the other colors, red and green, just the contrary of what one observes in the atrophy of the papilla (optic disk) of ataxics.

M. Galezowski has described a peculiar form of dyschromatopsia, which is due to aphasia. These patients confound one color with another, not because they are unable to see, but because they cannot call their names.

In diabetes and lead-poisoning there exists very marked adventitious color blindness, which can declare itself from day to day. In the presence of so many accidents on account of pathological dyschromatopsias, one should ask himself whether railroad employees ought not to be subjected, not to a single examination upon their entry into the service, but to examinations more or less frequent, and which permit, by being made at fixed epochs, of controlling the state of their vision.—*Rev. de Therapeutique*—*Western Med. Reporter.*

